# The protective effect of peroxiredoxin II on oxidative stress induced apoptosis in pancreatic β-cells

**DOI:** 10.1186/2045-3701-2-22

**Published:** 2012-06-18

**Authors:** Fang Zhao, Qinghua Wang

**Affiliations:** 1 Division of Endocrinology and Metabolism, the Keenan Research Centre in the Li Ka Shing Knowledge Institute, St. Michael's Hospital, 209 Victoria Street, Room 414, Toronto, ON, Canada, M5B 1T8; 2 Department of Physiology, University of Toronto, Toronto, ON, Canada

**Keywords:** β-cells, Apoptosis, Peroxiredoxin 2, Oxidative stress

## Abstract

Excessive loss of pancreatic β-cells, mainly through apoptosis, contributes to the development of diabetic hyperglycemia. Oxidative stress plays a major role in the process of β-cell apoptosis due to low expression level of endogenous antioxidants in the β-cells. Peroxiredoxins (PRDX) are a family of peroxide reductases which uses thioredoxin to clear peroxides. Several members of PRDX have been found in β-cells and recent studies suggested that these antioxidant enzymes possess protective effects in β-cells against oxidative stress mediated apoptosis. In this study, we aimed to investigate the role of PRDX2 in modulating β-cell functions. We detected the expression of PRDX2 both at the transcript and protein levels in the clonal β-cells INS-1 and MIN6 as well as rodent islets. Western blot showed that treatment of MIN6 β-cell line with proinflammatory cytokines, palmitic acid or streptozotocin dose- or time-dependently increased apoptosis, which was associated with reduced endogenous expression levels of PRDX2. To examine the role for PRDX2 in the apoptotic stimuli-induced β-cell apoptosis, we used plasmid overexpression and siRNA knockdown strategies to investigate whether the elevation or knockdown of PRDX2 affects stimuli-induced apoptosis in the β-cells. Remarkably, overexpression of PRDX2 in MIN6 cells significantly attenuated the oxidative stresses mediated apoptosis, as evaluated by cleaved caspase 3 expression, nuclear condensation and fragmentation, as well as FACS analysis. Conversely, attenuation of PRDX2 protein expression using siRNA knockdown exaggerated the cell death induced by proinflammatory cytokines and palmitic acid in the MIN6 cells. These results suggest that PRDX2 may play a protective role in pancreatic β-cells under oxidative stress.

## Introduction

Excessive loss of pancreatic β-cell mass, mainly due to apoptosis, is a major cause in the development of diabetic hyperglycemia in both type 1 and type 2 diabetes mellitus [1]. β-cell apoptosis is initiated by a variety of stimuli such as inflammatory cytokines, chronic hyperglycemia and hyperlipidemia [2,3] and downstream effects such as endoplasmic reticulum stress [4] and mitochondrial dysfunction [5]. Oxidative stress plays a permissive role in the process of apoptosis leading to cell destruction in many types of cell lineages [6,7]. Particularly the β-cells are more susceptible to oxidative stress due to the fact that they express major antioxidants such as superoxide dismutase, catalase and glutathione peroxidase at low levels [1,8,9]. In the pancreatic islets, superoxide dismutase expression is 30-40% compared with that of the liver, glutathione peroxidase expression is 15%, and catalase expression cannot be detected [10].

At the cellular level, oxidative stress-mediated β-cell apoptosis can result from an imbalance between reactive oxygen species (ROS) generation and its clearance by antioxidants [9]. It has been demonstrated that proinflammatory cytokines induced β-cell apoptosis is mediated through elevation of ROS in the mitochondria via altered electron transport chain action [11], and increased nitric oxide (NO) production via activation of inducible nitric oxide synthase (iNOS) [12]. The process is known to be involved with activation of the nuclear factor-κB (NF-κB) and the c-Jun N-terminal kinase (JNK/SAPK) or the FAS-FAS ligand pathways [13]. Induction of ROS is found to be multilateral. Long chain saturated non-esterized fatty acids (NEFA) such as palmitic acid (PA) induces ROS production in the mitochondria through the electron transport chain [11,14]. The long chain saturated NEFAs could also directly interact with respiratory chain proteins and increase the oxygen radicals [15]. Streptozotocin (STZ) is a toxic agent that causes β-cell death via DNA alkylation causing strand breaks and induction of apoptosis [16], and local injection of STZ can produce oxidative stress *in situ* causing tissue or organ dysfunction [17]. Previous studies suggested that STZ can increase production of oxygen radicals [18], and induction H_2_O_2_ and DNA fragmentation [19] in the pancreatic β-cells [16,20].

Peroxiredoxins (PRDX) are a family of antioxidant enzymes which is capable of metabolizing hydrogen peroxide [21]. PRDXs are thioredoxin-specific antioxidants first identified in yeast and are found in archea, prokaryotes as well as eukaryotes [22]. To date, six members of PRDXs have been found to be expressed in mammalian cells, as well as in the pancreatic β-cells [23].

Previous studies have suggested that PRDX2 can regulate many cellular functions such as cell proliferation and differentiation [24,25]. Through the clearance of H_2_O_2,_ PRDX2 also play critical role in the modulation of cell survival [26]. A recent study demonstrated that attenuation of PRDX2 inhibited proliferation and induced apoptosis in granulosa cells. This was achieved through the modulation of the NF-κB/iNOS pathway [27]. In primary cortical neurons, overexpression of PRDX2 protected against apoptosis through the suppression of the apoptotic ASK-1 signalling pathway [28,29]. PRDX2 is found to be relatively highly expressed in the pancreatic islet, i.e. with up to 3 fold higher compared with the liver [30]. However, the biological functions of PRDX2 in the pancreatic β-cells are not known. In this study, we investigated PRDX2 expression and its role in modulating β-cell survival and death in the mouse β-cell line MIN6.

## Results

### Expression of PRDX2 in pancreatic β-cells

**Figure 1:**
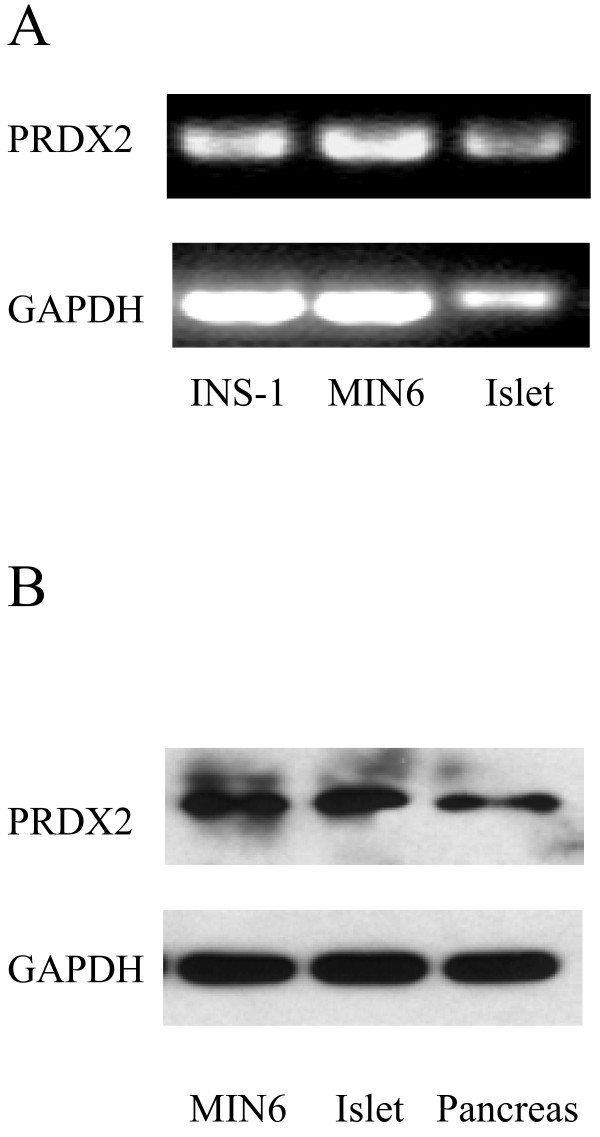
**PRDX2 is detected in the pancreatic β-cell lines and islet.** RT-PCR performed on RNA extracted from INS-1, MIN6, and mouse Islet (**A**). Western blot performed on protein extracted from MIN6, isolated mouse islets, and,mouse pancreas (**B**).

It has been previously reported that PRDX2 is expressed in variety of cells and tissues [31]. To determine whether PRDX2 is expressed in pancreatic β-cells, we performed RT-PCR and Western blot analysis. As shown, both PRDX2 transcripts and proteins are detected in clonal insulin secreting cell lines, isolated islets or pancreatic tissues (Figure 1A1B).

### Oxidative stress induced apoptosis and decreased PRDX2 expression in β-cells

**Figure 2:**
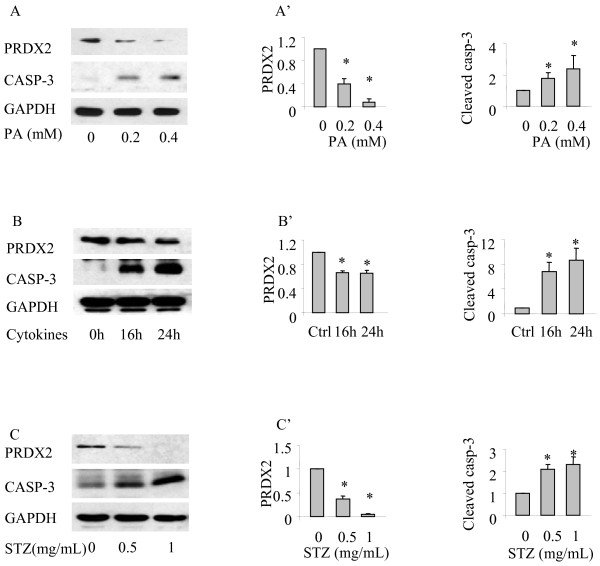
**PRDX2 expression in β-cells is decreased during oxidative stress induced apoptosis.** Western blot performed on proteins extracted from MIN6 cells treated with palmitic acid for indicated concentrations (**A**), or cytokine cocktail for indicated duration (**B**), or STZ for indicated concentrations (**C**). Quantitative analysis of relative PRDX2 expression and relative cleaved caspase 3 expression in MIN6 cells with SEM (A’-C’). n = 3 *p < 0.05.

To examine the PRDX2 expression during the process of oxidative stress-mediated apoptosis in the β-cells, MIN6 cells were treated with or without the oxidative stress agents PA, cytokines or STZ at indicated concentrations and for the indicated times. Cell lysates were subjected to Western blot analysis using relevant antibodies. As shown, incubation of MIN6 cells with tested oxidative stress inducers resulted in significant apoptosis as determined by increased cleaved form of caspase-3 levels, which was associated with decreased levels of PRDX2 expression (Figure 2A-C). Densitometry analysis of the Western blots showed that the reduction of PRDX2 levels in the β-cells treated with various oxidative stress agents were statistically significant (Figure 2A’-2 C’, *p < 0.05, n = 3). These results indicate that endogenous PRDX2 expression level is reduced upon oxidative stress in the pancreatic β-cells.

### Elevation of PRDX2 expression protects against oxidative stress-induced apoptosis in the β-cells

**Figure 3:**
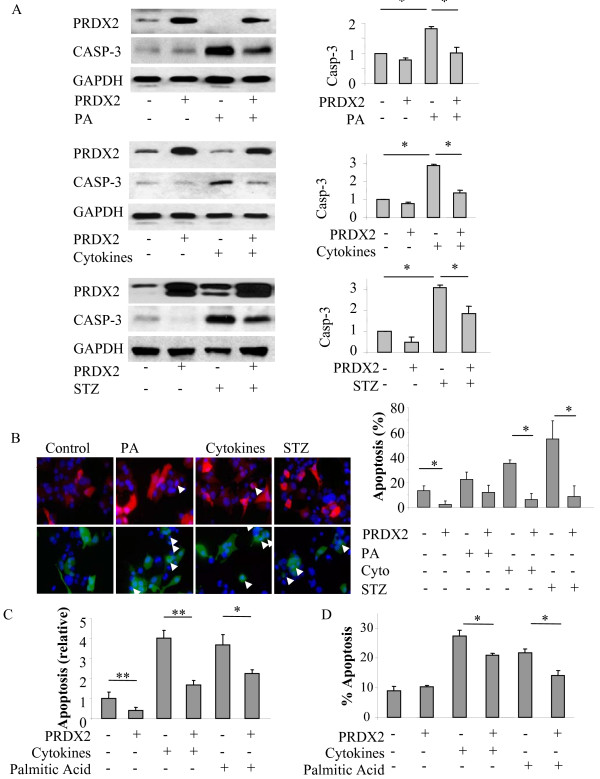
**Elevation of PRDX2 expression protects against oxidative stress induced apoptosis in the β-cells.** Western blot of MIN6 cells transfected with PRDX2 or empty vector (EV) and treated with indicated agents (**A**). Quantitative analysis of cleaved caspase 3 expression with SEM, n = 4 *p < 0.05. Immunocytochemistry of MIN6 cells with transfected with PRDX2 (red) or EV (green) and treated with indicated agents. Nuclei of cells were stained with DAPI (blue) (**B**). Quantitative analysis of abnormal nuclear morphology used as an indicator of apoptosis with SEM. n = 3 *p < 0.05. Fluorescence assisted cell sorting (FACS) of MIN6 cells transfected with PRDX2 or EV and treated with indicated agents. Cells were than treated with propidium iodide (PI) only or Annexin V FITC and PI and subjected to FACS. Absorbance in the FL-1 and FL-2 channel was recorded for a minimum of 10,000 events per sample to indicate Annexin V and PI positive cells respectively. (**C**) Quantitative analysis of FL-2 absorbance used as an indicator of cell death with SEM. n = 4, *p < 0.05, **p < 0.01. (**D**) Quantitative analysis of both FL-1 and FL-2 absorbance used as an indicator of apoptosis with SEM. n = 3, *p < 0.05.

To determine the role of PRDX2 in the process of oxidative stress-induced β-cell apoptosis, we investigated whether overexpression of PRDX2 would protect against the apoptosis induced by oxidative stress stimulating agents. Western blotting showed that elevation of PRDX2 by transfection displayed a significant reduction in apoptosis induced by either PA, or the cytokines or STZ in the transfected MIN6 cells, as evaluated by the levels of cleaved caspase-3 (Figure 3A, *p < 0.05, n = 3).

Apoptotic assays were conducted to examine the apoptosis at the cellular level using DAPI nuclear staining. We found that the three oxidative stress agents produced profound apoptosis as determined by condensed or fragmented nuclei in the mock transfected MIN6 cells, which was significantly reduced in the cells overexpressing PRDX2 (Figure 3B, *p < 0.05, n = 3). Consistently, FACS using propidium iodide (Figure 3C) and Annexin V FITC (Figure 3D) also demonstrated that the oxidative stress induced β-cell apoptosis was remarkably reduced in the cells overexpressing PRDX2 (*p < 0.05, n = 3-4).

These results suggest that elevation of PRDX2 protein expression can attenuate the apoptosis induced by PA, or the cytokines or STZ in MIN6 cell line, which is suggestive of the protective effects of PRDX2 in the β-cells.

### PRDX2 knockdown exaggerated oxidative stress-induced apoptosis in the β-cells

**Figure 4:**
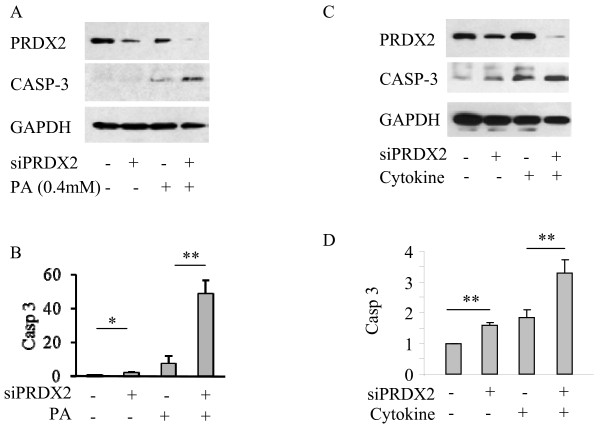
**Knockdown of PRDX2 expression exaggerates oxidative stress induced apoptosis in the β-cells.** Western blot performed on cell lysate of MIN6 cells with PRDX2 knockdown via siRNA transfection or scrambled control followed with treatment of 0.4 mM palmitic acid or proinflammatory cytokine cocktail for 24 hrs (**A, C**). Quantitative analysis of cleaved caspase 3 expression with SEM, (**B, D**) n = 3 *p < 0.05.

We further conducted knockdown studies using siRNA method to verify the protective role of PRDX2 in the β-cells. Transfection of MIN6 cells with PRDX2 siRNA resulted in reduction of >80% PRDX2 in the MIN6 cells (Figure 4A, 4C). Western blot analysis showed significantly exaggerated apoptosis induced by cytokines or palmitic acid in the MIN6 cells transfected with siRNA targeting PRDX2 but not the cells transfected with scrambled siRNA (Figure 4B, 4D, *p < 0.05, n = 3). These results suggested that attenuation of PRDX2 by siRNA knockdown removes the protective effects of PRDX2 and increases apoptosis in the β-cells.

## Discussion

Excessive apoptosis of the pancreatic β-cell causing loss of β-cell mass is a major contributor to the initiation and progression of diabetes [12,32,33]. β-cell apoptosis can be induced by multiple stimuli including proinflammatory cytokines and long chain NEFAs in both type 1 and type 2 diabetes [9,34]. In rodent models of β-cell injury, drugs like STZ can induce various ROS, and act partially through oxidative stress to induce β-cell apoptosis that leads to the loss of β-cell mass [16,19]. Previous pre-clinical and clinical studies demonstrated that the elevation of ROS was associated with decreased antioxidant capacity in the islet β-cells in type 1 and type 2 diabetic subjects [35,36]. Islet β-cells expresses low levels of major antioxidants therefore they are particularly vulnerable to the detrimental effects of ROS mediated cellular injury compared to other cell types [1,10,37]

Detection of PRDX2, both at transcript and protein levels in the mouse pancreas, isolated mouse islets and the clonal MIN6 cells is consistent with previous findings that demonstrated the presence of PRDX2 in the INS-1 cells and rodent islets [37]. However the biological function of PRDX2 in the β-cells was not investigated. We aimed to examine whether PRDX2 has protective effects against β-cell apoptosis that occurs under oxidative stress conditions. In MIN6 cells, we found that the apoptosis induced by proinflammatory cytokines, palmitic acid, or STZ was associated with significantly decreased endogenous PRDX2 expression. To further study the potential role for PRDX2 in the β-cells during oxidative stress mediated apoptosis, we overexpressed PRDX2 in MIN6 cells. We found that the elevation of PRDX2 expression through transfection protected against oxidative stress mediated apoptosis. Furthermore, reduction of PRDX2 expression through siRNA knockdown strategy exaggerated the oxidative stress stimuli- induced apoptosis. These findings suggest that PRDX2 may act as an important survival factor in pancreatic β-cells.

In the β-cells, proinflammatory cytokine [38] or palmitatic acid [39] induced β-cell apoptosis is partially mediated through the activation of NF-κB pathway. In a recent study, it has been reported that PRDX2 can prevent oxidative stress induced apoptosis through modulating NF-κB activity in granulosa cells [27]. It has been shown that in embryonic fibroblast derived from mice lacking PRDX2 exhibited increased NF-κB activities [40]. These findings suggest that PRDX2 may prevent apoptosis through inhibiting NF-κB. While the precise mechanism requires further investigation, it is likely that PRDX2 use the similar mechanism to regulate survival or apoptosis in the β-cells. This notion is supported by a recent study which demonstrated that PRDX3, a peroxiredoxin member primarily localized in mitochondria, is able to protect β-cell from stress due to accumulation of hydrogen peroxide or the induction of iNOS by STZ or inflammatory cytokines [41]. iNOS induction that activates apoptotic pathways through the activation NF-κB has been well documented [42,43].

Our observations are consistent with previously findings by others that gene expression of PRDXs is adjustable by oxidative stress agents. Of note, however, high glucose induced cellular stress has no obvious effects on other antioxidants in rat pancreatic islets or in clonal β-cell line [44]. β-cell apoptosis induced by long chain saturated NEFA, such as palmitic acid, is mediated through oxidative endoplasmic reticulum (ER) stress [45-47]. Previous studies showed that long chain NEFA induced ER stress is partially mediated through the activation of the NF-κB pathway [48] and iNOS activation [49]. In accord with this, a recent study demonstrated that palmitate induced apoptosis in the β-cells was attenuated by anti-diabetic agent pioglitazone, partially through the suppression of NF-κB [50]. It is presumably that the anti-apoptotic effects of PRDX2, particularly in preventing palmitic acid induced apoptosis in the β-cells is partially through suppression of NF-κB. Further investigation is warranted to dissect the precise signalling events conveying PRDX2 action to survival effectors.

## Conclusions

β-cells are particularly susceptible to oxidative stress due to low expression level of endogenous antioxidants in these cells. We have identified that PRDX2 is highly expressed in the β-cells. We fund that challenge INS-1 cells with cytotoxic cytokines, palmitic acid or streptozotocin resulted in remarkable apoptosis associated with reduced PRDX2 expression. Overexpression or knockdown studies by plasmid or siRNA transfection in the clonal β-cells in the presence of various oxidative stimuli suggested that PRDX2 may play a protective role in modulating pancreatic β-cell function. These findings may provide a better understanding on the regulatory nature of β-cell survival and apoptosis, facilitating our efforts to identify valid targets to prevent β-cell death in the development of diabetes.

## Methods

### Pancreatic islets and cell line

Mouse insulinoma cell line MIN6, passage 50–70 (from Dr. Maria Rozakis Adcock, University of Toronto) were maintained in RPMI 1640 medium (Invitrogen, Burlington, ON, Canada) containing FBS (10% v/v), 100 units/ml penicillin G sodium, 100 μg/ml streptomycin sulphate, 55 mg/500 ml sodium pyruvate, 1.14 g/500 ml HEPES, and 1.7 μL/500 ml β-mercaptoethanol at 37 °C in an atmosphere of humidified air (95%) and CO_2_ (5%). In studies involving serum-starvation, serum was replaced by 0.1% BSA in serum free RPMI 1640 (SFM). INS-1 cells were maintained as described previously [51,52]. Rat islets were isolated from male Sprague–Dawley rats (weight 150–200 g) (Charles River Canada, Montreal, QC, Canada) as described [53,54]

### Constructs used in this study

pCMVsport6-PRDX2 vector was purchased from Open Biosystems (Huntsville, AL, USA). siGENOME smartpool siRNA was purchased from Dharmacon RNAi technologies (Chicago, IL, USA). Scrambled siRNA was purchased from Ambion (Austin, TX, USA)

### Oxidative stress agents and treatment

Palmitic acid was dissolved in serum free RPMI 1640 medium containing 1% fatty acid-free BSA (Sigma Aldrich, Oakville, ON, Canada) through an incubation of the mixture (225 rpm) at 37 °C for 3 hours allowing for binding of PA with BSA. A cytokine cocktail mixture (IL-1β, 10 ng/ml; TNF-α, 50 ng/ml, and IFN-γ, 50 ng/ml, R&D Systems, Minneapolis, MN, USA) was prepared in SFM. Streptozotocin (STZ) (Sigma Aldrich) dissolved in the SFM was freshly prepared prior to treatment. MIN6 cells seeded at 80% confluency in 12 well plates the night before were serum starved for 1 hour then treated with PA or the cytokines for indicated times, or STZ for 4 h. Cells were either fixed or lysed for subsequent experimentation.

### Overexpression and PRDX2 knockdown studies

Overexpression of PRDX2 in MIN6 cells was achieved through transfection of the plasmid encoding PRDX2 (pCMVsport6-PRDX2, 1.6 μg per well of 12 well plate) or mock transfection as control. Knockdown of PRDX2 in MIN6 cells was accomplished through transfection of small interfering RNA (siRNA) targeting mouse PRDX2 (siGENOME SMARTpool, 40pmol per well of 12 well plate) or scrambled siRNA as control. The day before transfection the MIN6 cells were seeded at 5 × 10^5^ per well in a 12-well plate. The cells were transfected at 80% confluency using lipofectamine 2000 according to manufacturer’s protocols. The transfected cells were allowed to grow in complete medium for 24 hours post transfection before treatment with stress agents.

### RT-PCR

Total RNA was extracted using TRIzol (Invitrogen) reagent according to the manufacturer's instructions. RT-PCR was performed using AffinityScript one-step RTPCR kit (Stratagene, Mississauga, ON, Canada). The primers used were: PRDX2, fwd: 5’ ATCCCTCTGCTTGCTGATGT 3’, and rev: 5’ TTGACTGTGATCTGGCGAAG 3’; GAPDH: fwd: 5’ TGCCACTCAGAAGACTGTGG 3’, and rev: 5’ TTCAGCTCTGGGATGACCTT 3’. The DNA was first denatured at 95 °C then annealed at 60 °C and extended at 72 °C. This was repeated for 30 cycles before a final extension at 72 °C for 10 minutes. The PCR product was ran on a 2% agarose gel and visualized by ethidium bromide.

### Western blot analysis

Cells and tissues were lysed in RIPA lysis buffer containing the protease inhibitors phenylmethylsulphonylfluoride (PMSF) (1 mol/l) and EDTA (1 mol/l), Na_3_VO_4_ (1 mol/l), and NaF (1 mol/l). Protein of 25 μg was resolved by SDS-PAGE, transferred to nitrocellulose membranes and probed by anti-PRDX2 (1:1000, ABCAM, Cambridge, MA, USA), GAPDH (1:20,000, ABCAM) or cleaved caspase-3 (1:1000, Cell Signalling Technology Inc., Danvers, MA, USA) visualized by enhanced chemiluminescence (GE Healthcare Bio-Sciences Corp., Piscataway, NJ, USA) as described previously [55]. The films were then scanned and the intensity of the bands was quantified using ImageJ (Research Services Branch, National Institute of Mental Health, Bethesda, Maryland, USA) [55].

### Immunocytochemistry

MIN6 cells grown on poly-L-lysine (Sigma) coated cover slips in 12 well plates were transfected with PRDX2, or GFP as control, using lipofectamine 2000 according to manufacturer’s protocol. 24 hours post transfection, the cells were serum starved (1 h) and treated with PA, the cytokine cocktail or STZ as indicated. Cells were fixed with 4% paraformaldehyde and blocked with 3% BSA in PBS containing 0.1% Triton X-100 at room temperature for 1 hour. The cells were then incubated with mouse monoclonal PRDX2 antibody (ABCAM, 1:100) and Alexa 555-conjugated anti-mouse IgG (Jackson Labs, 1:500), and visualized with Nikon Fluorescence microscope (Eclipse TE 200). 300–450 transfected cells identified by GFP were counted per sample.

### Detection of apoptosis

Apoptosis of MIN6 cells was evaluated by DAPI-nuclear staining based on typical chromatin condensation and fragmentation. The number of transfected apoptotic cells was counted and compared to the total number of transfected cells, a minimum of 300 cells were counted for each group.

Fluorescence-activated cell sorting (FACS) was used to quantify propidium iodide (PI) and Annexin V FITC stained cells transfected with or without PRDX2 that were treated with palmitic acid, cytokines as indicated. FACS-Calibur flow cytometer (Becton-Dickinson Biosciences, Mississauga, ON, Canada) was used and the results were analysed using FCS Express version 3 (De Novo Software, Los Angeles, CA, USA). Fluorescence in the FL1 channel (log green fluorescence, 485/535 nm) for Annexin V FITC and FL2 channel (log red fluorescence, 650 nm) for PI were acquired and recorded, using logarithmic scales, for a minimum of 10,000 events per sample.

### Statistical analysis

All data were presented as mean±SEM. Statistical analysis was performed using unpaired two-tailed Student’s *T*-test or ANOVA with Bonferroni’s multiple comparisons post hoc test where appropriate. A p-value of less than 0.05 was considered significant.

## Competing interests

The authors declare that they have no competing interests.

## Authors’ contributions

FZ performed all experiments and wrote the manuscript. QW designed the overall study and wrote the manuscript. Both authors read and approved the final manuscript.
